# Single-Cell Metabolomics in Hematopoiesis and Hematological Malignancies

**DOI:** 10.3389/fonc.2022.931393

**Published:** 2022-07-13

**Authors:** Fengli Zuo, Jing Yu, Xiujing He

**Affiliations:** Laboratory of Integrative Medicine, Clinical Research Center for Breast, State Key Laboratory of Biotherapy, West China Hospital, Sichuan University and Collaborative Innovation Center, Chengdu, China

**Keywords:** single-cell metabolomics, metabolic reprogramming, hematopoiesis, hematological malignancies, glucose metabolism, amino acids metabolism, lipid-related metabolism

## Abstract

Aberrant metabolism contributes to tumor initiation, progression, metastasis, and drug resistance. Metabolic dysregulation has emerged as a hallmark of several hematologic malignancies. Decoding the molecular mechanism underlying metabolic rewiring in hematological malignancies would provide promising avenues for novel therapeutic interventions. Single-cell metabolic analysis can directly offer a meaningful readout of the cellular phenotype, allowing us to comprehensively dissect cellular states and access biological information unobtainable from bulk analysis. In this review, we first highlight the unique metabolic properties of hematologic malignancies and underscore potential metabolic vulnerabilities. We then emphasize the emerging single-cell metabolomics techniques, aiming to provide a guide to interrogating metabolism at single-cell resolution. Furthermore, we summarize recent studies demonstrating the power of single-cell metabolomics to uncover the roles of metabolic rewiring in tumor biology, cellular heterogeneity, immunometabolism, and therapeutic resistance. Meanwhile, we describe a practical view of the potential applications of single-cell metabolomics in hematopoiesis and hematological malignancies. Finally, we present the challenges and perspectives of single-cell metabolomics development.

## Introduction

Metabolism consists of a series of biochemical reactions that occur within a living organism to maintain life. As genetic or non-genetic alterations, tumor cells rewire metabolic pathways to adapt to their rapid growth and proliferation. Metabolic reprogramming of cancer cells is now deemed one of the hallmarks of cancer (1). Increasing evidence suggests that dysregulated cell metabolism facilitates tumor initiation, progression, metastasis, and drug resistance. The metabolic alterations of cancer cells are mainly reflected in the increase in glucose and glutamine uptake and fatty acid metabolism, which are crucial for promoting the rapid synthesis of nucleotides, proteins, and lipids, meeting energy requirements and maintaining redox homeostasis (2–4). Moreover, metabolic alterations also modulate cell signaling pathways and post-translational modifications (PTMs) (5). Metabolites can serve as signaling molecules that directly affect both pro-inflammatory and anti-inflammatory outcomes (6–8). Emerging evidence suggests that metabolic regulation of PTMs on DNA and histones impacts gene expression (9, 10). Furthermore, metabolic enzymes have been reported to have ‘moonlighting’ functions as RNA-binding proteins (11). Metabolic alterations in cancer are triggered by various mechanisms that instigate signaling pathways and regulate the expression of metabolism-related genes (3).

Alterations in metabolic processes vary from cancer to cancer, as nutrient availability, oncogenic activation, proliferative state or microenvironment are spatially and temporally heterogeneous. As a result, each type of cancer cell has distinct needs in terms of energy and biomass production (12–14). When analyzing the metabolism of different hematological malignancies, the heterogeneity between them should also be considered.

A growing number of studies regard cancer as a kind of metabolic diseases, as do hematological malignancies. Hematological malignancies can be classified as leukemia, myeloma, and lymphoma and are often deadly. The most common hematological malignancies include acute myeloid leukemia (AML), chronic myeloid leukemia (CML), acute lymphoblastic leukemia (ALL), chronic lymphocytic leukemia (CLL), multiple myeloma (MM), Hodgkin lymphoma (HL), and non-Hodgkin lymphoma (NHL). Aberrant metabolism and metabolic reprogramming play important roles in the pathogenesis of hematologic disorders. The metabolic characteristics of leukemia cells are usually different from those of their normal counterparts, manifested by increased glycolysis, glutaminolysis, and lipogenesis. Metabolic differences provide new therapeutic targets to overcome hematological malignancies. Moreover, metabolic reprogramming contributes to an immunosuppressive microenvironment, increasing the probability of resistance to anticancer therapies. Recurrence and refractory always exist in patients with hematologic malignancies, and long-term overall survival remains unsatisfactory. Newer and more sophisticated therapeutic approaches are imperative. Metabolic therapies alone or in combination with other treatment regimens, such as immunotherapy, targeted therapy, and chemotherapy, bring new opportunities for patients with hematologic malignancies.

**Figure 1:**
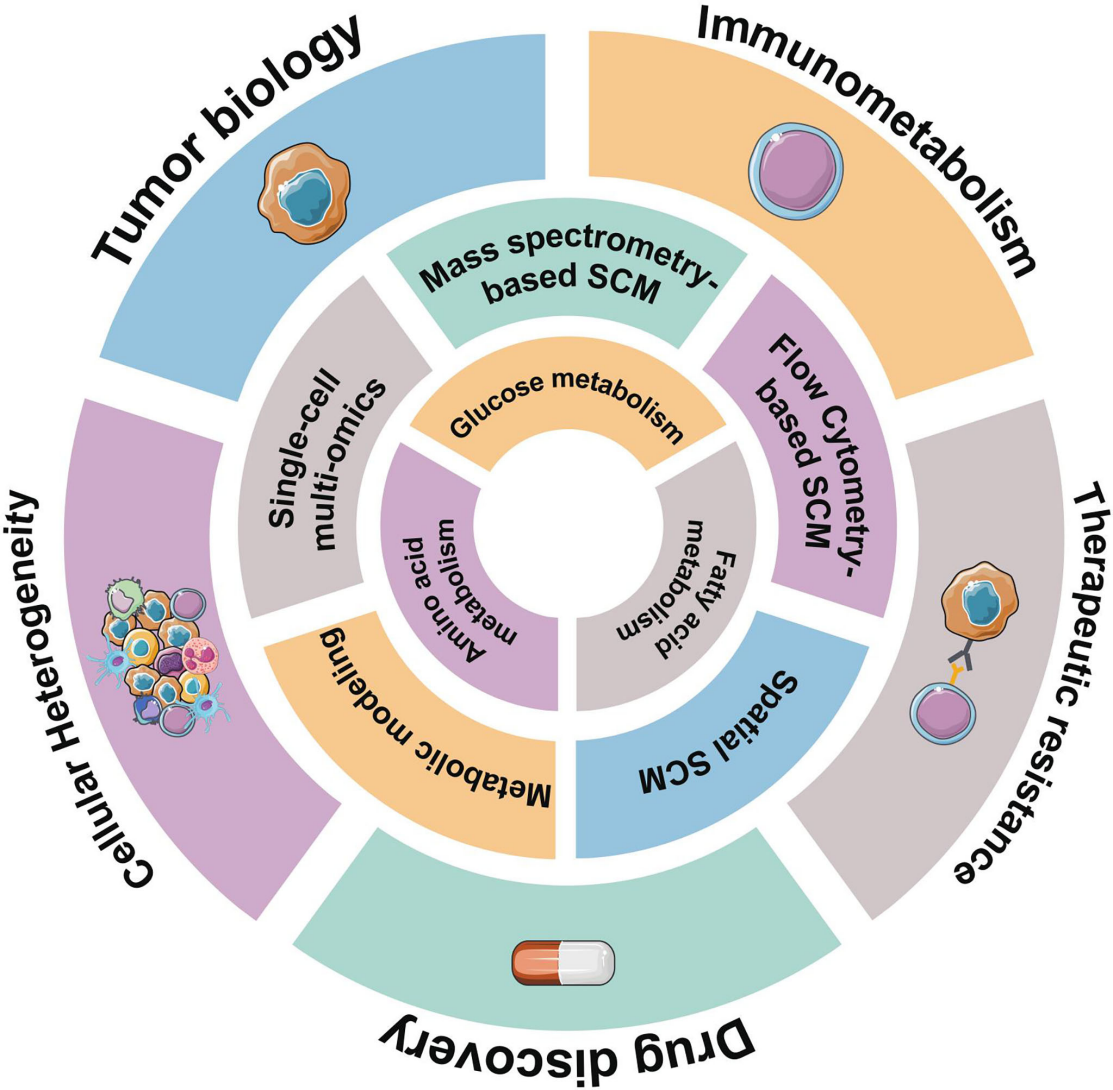
Overview of representative single-cell metabolomics methods for metabolic profiling. Hematological malignancies have abnormal metabolic characteristics, reflected in glucose metabolism, amino acids metabolism, and lipid-related metabolism. Some emerging single-cell metabolomics (SCM) techniques can help us better understand metabolic signatures at the single-cell level and provide unprecedented insights into hematological malignancies.

Metabolomics provides the best view of biological phenotypes by profiling changes in endogenous metabolites. Insights into the role of metabolic reprogramming in tumor biology have largely been accomplished by bulk metabolic analysis techniques. However, bulk analyses neglect intratumoral heterogeneity, so the mechanisms underlying critical disease events of hematological malignancies remain obscure, such as treatment resistance and clonal evolution. Propelled by a set of recent technological advances in single-cell metabolomics, new insights into tumor metabolism are rapidly emerging, which are often not available on other omics layers (**Figure 1**). Single-cell metabolomics technologies will provide an understanding of hematological malignancies at unprecedented depth and reveal new insights into the pathogenesis of hematologic malignancies.

In this review, we summarize the unique metabolic characteristics of hematologic malignancies. Then, we illustrate the research progress of single-cell metabolomics technology. Applications and potential of single-cell metabolomics in hematopoiesis and hematologic malignancies are discussed. Finally, we present the challenges and perspectives of single-cell metabolomics development. This review will provide a clear navigation of numerous single-cell metabolomics technologies and strategies.

## Unique Metabolic Properties of Hematologic Malignancies

### Glucose Metabolism

**Figure 2:**
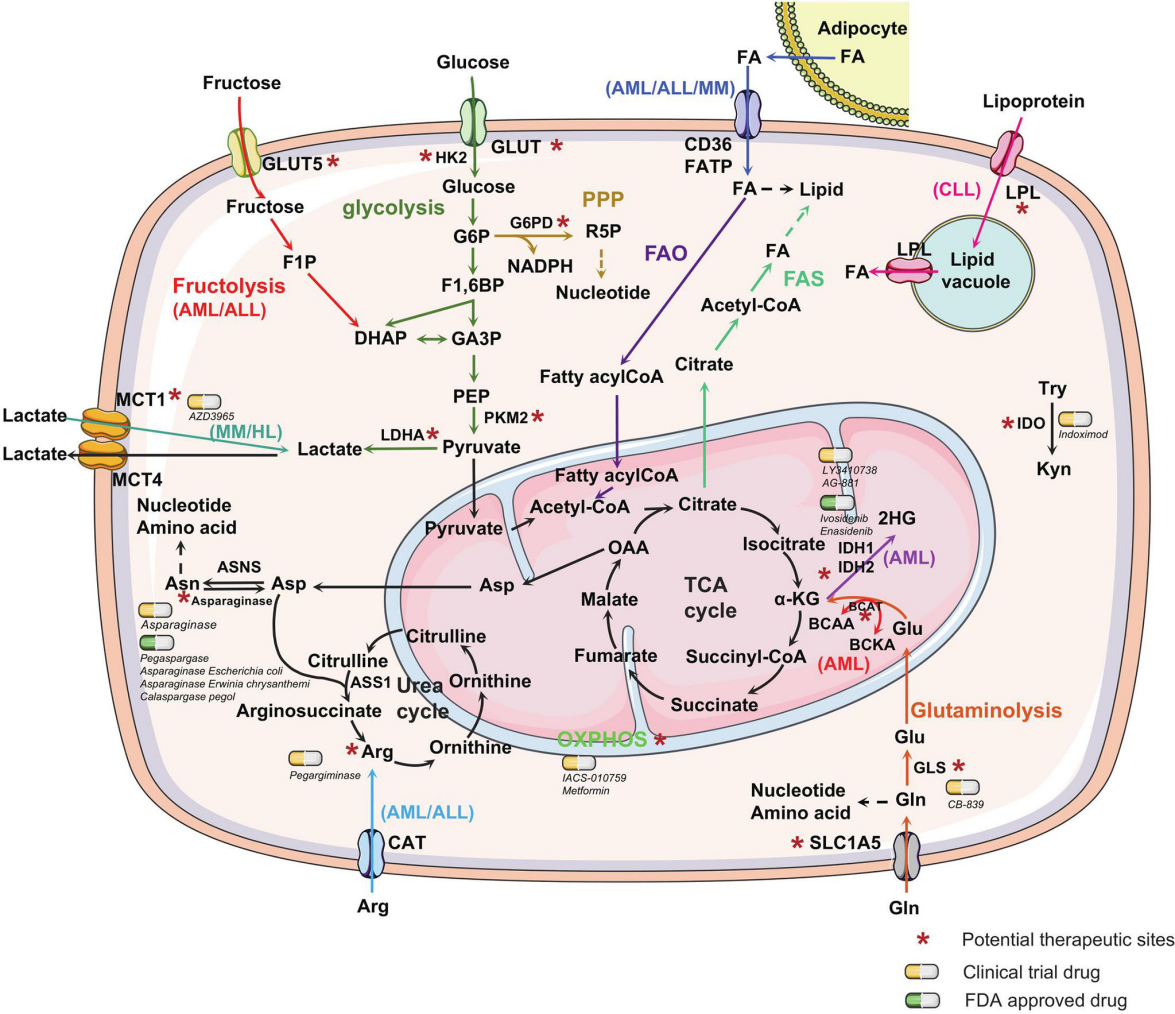
Metabolic alterations in hematological malignancies. Metabolic alterations in hematological malignancies are mainly reflected in the increase in glucose and glutamine uptake and fatty acid metabolism to promote the rapid synthesis of nucleotides, proteins, and lipids. Different metabolic pathways are marked with distinct colors. Unique metabolic characteristics exhibited in one or a few types of hematological malignancies are labeled. Some special enzymes or reaction processes in the metabolic process, which can be used as potential therapeutic targets, are marked with an asterisk. The yellow capsules represent the drugs in clinical trials, whereas the green capsules represent FDA-approved drugs targeting the metabolic process of hematological malignancies. The dashed line with arrow represents anabolism. GLUT, glucose transporter; HK2, hexokinase2; G6P, glucose-6-phosphate; G6PD, Glucose-6-phosphate dehydrogenase; R5P, ribose-5-phosphate; PPP, pentose phosphate pathway; NADPH, nicotinamide adenine dinucleotide phosphate; F1,6BP, fructose-1,6-biphosphate; GA3P, glyceraldehyde 3-phosphate; DHAP, dihydroxyacetone phosphate; F1P, fructose-1-phosphate; PEP, phosphoenolpyruvate; PKM2, pyruvate kinase M2; FA, fatty acid; FATP, fatty acid transport protein; OAA, oxaloacetate; α-KG, α-ketoglutarate; Asp, Asparagine; Asn, asparagine; ASNS, asparagine synthetase, ASS1, arginine succinate synthase-1; Arg, arginine; CAT, cationic amino acid transporters; BCAA, branched-chain amino acid; BCKA, branched-chain Keto acid; IDH, isocitrate dehydrogenases; 2HG, 2-hydroxyglutarate; Glu, Glutamate; Gln, glutamine; GLS, glutaminase; Try, tryptophan; Kyn, kynurenine; IDO, indoleamine 2,3-dioxygenase, LPL, lipoprotein lipase; MCT, monocarboxylate transporter; LDHA, lactate dehydrogenase A; FAO, fatty acid oxidation; FAS, fatty acid synthesis; TCA, tricarboxylic acid cycle.

**Figure 3:**
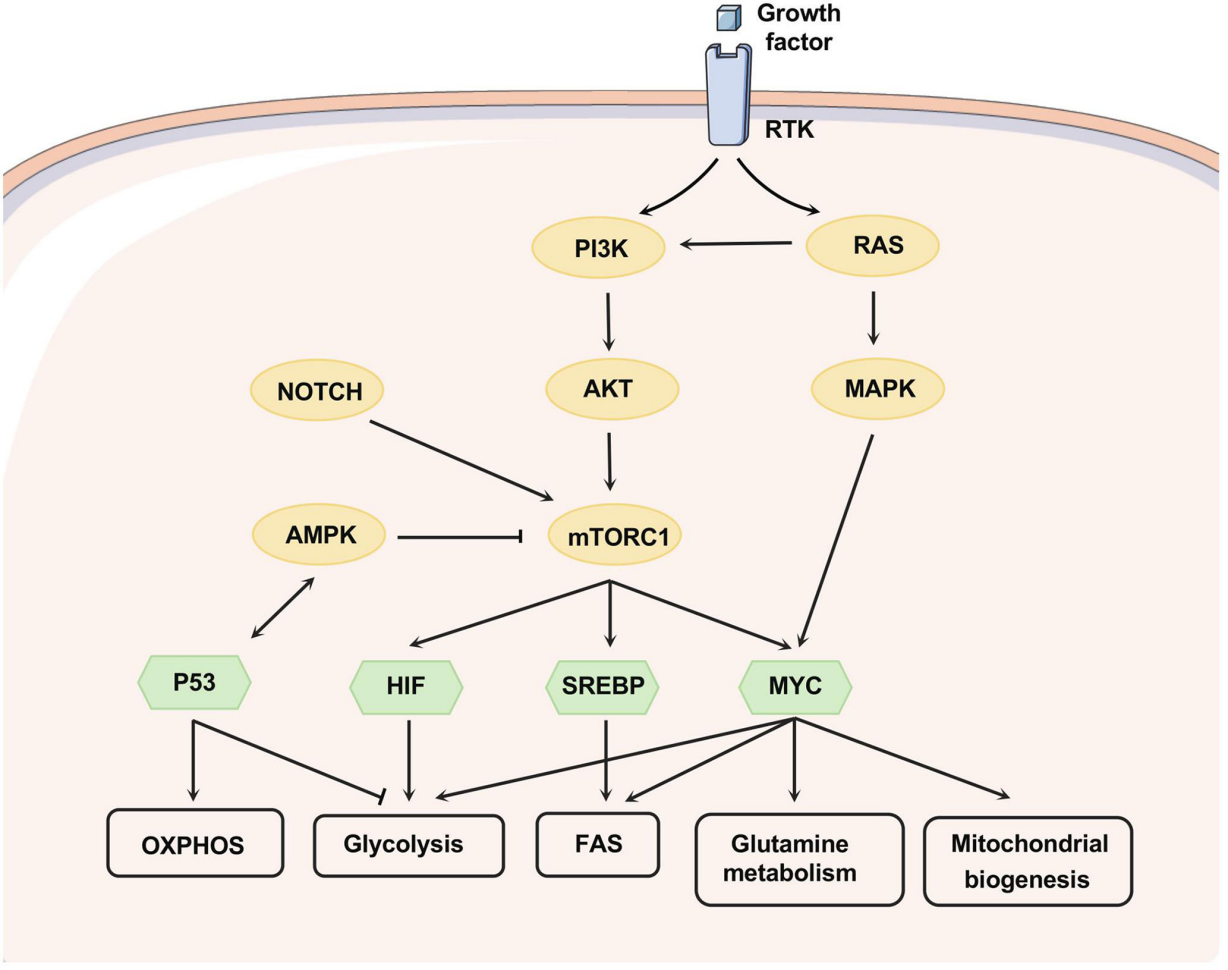
Signaling pathways that regulate metabolism. Growth factors affect metabolism by activating RTKs, and the PI3K/AKT axis can be activated as downstream of RTK and RAS. mTORC1, downstream of PI3K/AKT, can be activated upon AKT-mediated phosphorylation or suppressed through AMPK-mediated phosphorylation. NOTCH1 signaling promotes the activation of PI3K-AKT-mTOR signaling. PI3K-AKT-mTOR signaling can alter metabolism by regulating multiple transcription factors, nutrient transporters, and phosphorylating metabolic enzymes. HIF, MYC, and SREBP-1 are downstream transcription factors of mTORC1 that promote glycolysis, glutamine metabolism, fatty acid synthesis, and mitochondrial biogenesis. MYC can collaborate with HIF to enhance the expression of genes involved in glucose uptake and glycolysis, including LDHA, pyruvate dehydrogenase kinase-1 (PDK-1), and HK-2. Apart from this, MYC targets function to enhance mitochondrial biogenesis and function, especially glutamine metabolism. MYC can induce glutamine transporters expression (e.g. SLC7A5 and SLC1A5) and increase the levels of glutaminase. SREBP as a downstream effector of mTORC1 can induce the expression of several key enzymes related to fatty acid and sterol biosynthesis, such as ATP citrate lyase (ACLY) and fatty acid synthase. Moreover, SREBP interacts with MYC to regulate lipogenesis and then promote tumorigenesis. P53 forms a positive feedback loop with AMPK to repress glycolytic activity and promote OXPHOS and PPP. Phosphoserine STAT3 binds to and activates the promoter of LPL.

The most common metabolic alteration in cancer is aerobic glycolysis. In the presence of oxygen, normal cells will take up glucose for respiration and continue with oxidative phosphorylation (OXPHOS), whereas some tumor cells are more likely to take glucose for glycolysis to rapidly produce ATP, anabolic intermediates and lactate (1), which is known as the Warburg effect. Lactate can promote tumor cell growth and metastasis by stimulating angiogenesis and acidifying the tumor microenvironment, and also cause local inflammatory responses (15–17). A high level of glucose consumption is a conserved characteristic of most hematological malignancies (**Figure 2**). The PI3K-AKT/mTOR signaling pathway activates the expression of downstream glycolytic genes, including *GLUT1, HK2, PFKFB3, LDHA*, *PKM2* and suppressors of the tricarboxylic acid (TCA) cycle such as *PDK (* 4), resulting in a shift in glucose utilization (**Figure 3**). Competitive glucose metabolism as a target boosts the emergence of novel therapeutic approaches for hematologic malignancies.

Cellular glucose uptake is mediated by transmembrane glucose transporters (GLUTs), and most hematological malignancies, such as AML, CML, B-ALL and MM, take up abundant glucose through overexpressed GLUT1. Studies have demonstrated GLUT1 as a therapeutic target for hematological malignancies (**Figure 2**) (18–22). MM cells also exhibit an unexpected dependence on GLUT4, GLUT8 and GLUT11. Myeloma cells exhibit reliance on GLUT4 for basal glucose consumption, maintenance of apoptotic effector Mcl-1 expression, growth, and survival, while GLUT8 and GLUT11 are required for proliferation and viability in myeloma (23). Likewise, 60% of CLL cells overexpressed GLUT4, facilitating glucose transport (24). The human immunodeficiency virus (HIV) protease inhibitor ritonavir has an off-target inhibitory effect on GLUT4 expression in CLL and MM, resulting in the reduction of cell viability (23, 24). Notably, GLUT1 is not increased in CLL cells, and CLL cells seem not to follow the Warburg effect (25, 26). Distinct signatures of glucose metabolism have been found in AML patients, which demonstrated prognostic value in cytogenetically normal AML patients (27). Internal tandem duplication (ITD) mutation in the Fms-like tyrosine kinase 3 gene (FLT3/ITD) causes a significant increase in aerobic glycolysis through AKT-mediated upregulation of mitochondrial hexokinase 2 (HK2) and renders leukemia cells highly dependent on glycolysis (28). The combination of the FLT3 tyrosine kinase inhibitor sorafenib and glycolytic inhibitor 2-deoxy-d-glucose (2-DG) enhanced cytotoxicity in AML (28).

Pyruvate kinase (PKM2) and lactate dehydrogenase A (LDHA) play important roles in the initiation, maintenance, and progression of CML and AML in mice, and deletion of PKM2 or LDHA results in significantly prolonged disease latency (29). LDHA inhibitor oxamate suppressed proliferation and induced apoptosis in T-ALL cell lines and primary T-ALL cells through the c-Myc-ROS and PI3K/AKT/GSK3β signaling pathways (29). In the myeloma microenvironment, lactate is also produced by stromal cells and then enters myeloma cells *via* monocarboxylate transporter 1 (MCT1). Lactate contributes to the survival of MM cells in autocrine or paracrine manners (30). Treatment with α-cyano-4-hydroxy cinnamate (CHC), a known inhibitor of MCT, dose-dependently induced cell death in MM cell lines and primary MM cells (31). HL cells are prone to undergo OXPHOS within mitochondria. Some non-cancer cells, such as tumor-associated macrophages (TAMs) with high glycolysis, could promote tumor growth in HL (32). HL tumor cells have high mitochondrial metabolism, high expression of MCT1, and uptake and utilization of lactate released from TAMs.

Leukemia cells show a marked dependence on the pentose phosphate pathway (PPP), which is a branch of glycolysis, to generate ribose-5-phosphate (R5P) for nucleic acid synthesis and NADPH for biosynthetic reactions and oxidative balance (**Figure 2**). In AML serum samples, the PPP intermediate D-ribose phosphate was reduced (27). Critical PPP genes were upregulated in 61% of patients with AML (33). Glucose-6-phosphate dehydrogenase (G6PD) is the first enzyme in the PPP pathway, whose inhibitor 6-aminonicotinamide (6AN) induces cytotoxicity against AML cells *in vitro* and *in vivo* (34). In addition to PPP, fructolysis is an alternative strategy to provide carbon intermediates for the glycolytic pathway in AML (35). AML cells can compensate for low glucose levels by upregulating fructose transporter GLUT5 (36). GLUT5 is upregulated in Philadelphia chromosome-positive ALL (Ph^+^ ALL), leading to imatinib resistance, thus targeting GLUT5 might be promising in Ph^+^ ALL patients (37, 38).

Pyruvate and some other fuel sources, such as glutamine, can enter the mitochondrial TCA cycle and undergo OXPHOS, which is an important reaction in mitochondria. Although aerobic glycolysis is the main metabolic mode of hematological malignancies, some of them also increase OXPHOS to gain energy and anabolic precursors. The examined untreated CLL patients exhibit a metabolic signature of oxidative stress (25), and OXPHOS is a predominant pathway in CLL for energy production (39). CLL cells have an increased mitochondrial number and mass, displaying heightened mitochondrial respiration, elevated levels of reactive oxygen species (ROS), and enhanced antioxidant capacity (25, 40–42). Metformin inhibits mitochondrial complex I, inducing apoptosis of quiescent CLL cells and inhibiting cell cycle entry (43). Activated 5’ AMP-activated kinase (AMPK) inhibits mammalian target of rapamycin complex 1 (mTORC1) while promoting oxidative metabolism and mitochondrial complex I activity, resulting in a decreased level of aerobic glycolysis in T-ALL cells (44). AML patient samples display an increased mitochondrial mass without a concomitant increase in respiratory chain complexes activity, which makes AML cells seem more susceptible to oxidative stress (45). Increased glycolysis and inefficient OXPHOS in AML patients may contribute to drug resistance (46). However, in different AML cell lines, NB4 cells tend to undergo glycolysis, while THP-1 cells are recognized to be dependent on OXPHOS (47). THP-1 cells are resistant to 2-DG treatment, while NB4 cells are sensitive to 2-DG treatment; the difference is that AMPK responds differently to 2-DG (47).

Isocitrate dehydrogenase (IDH) catalyzes the decarboxylation of isocitrate to α-KG, however, mutant IDH1 and IDH2 reduce α-KG to 2-hydroxyglutarate (2HG), which could alter the epigenetic landscape of leukemic progenitors (48, 49). IDH mutations are prone to occur in AML (50, 51), and the FDA-approved drugs ivosidenib and enasidenib have been identified as small molecules, targeting IDH1 and IDH2 in AML, respectively. Furthermore, vorasidenib (AG-881) and LY3410738 are under investigation in phase I trials for the treatment of AML patients with IDH1 and/or IDH2 mutation (52, 53).

### Amino Acids Metabolism

Apart from glucose, cancer cells rely heavily on glutamine to obtain the necessary energy and building blocks to survive and proliferate. Glutamine serves as a carbon source for the replenishment of TCA cycle intermediates and a nitrogen source for the biosynthesis of nucleotides and amino acids. Glutamine is converted to glutamate by glutaminase (GLS). Almost all hematological malignancies depend on glutamine metabolism, and targeting glutamine metabolism has been proven to have therapeutic potential in the treatment of hematological malignancies (40, 54–57). For example, the glutaminase inhibitor CB-839 inhibits glutathione (GSH) production, induces mitochondrial reactive oxygen species (mitoROS) and causes apoptosis in AML and ALL (57). Knockdown of the glutamine transporter SLC1A5 inhibits glutamine uptake, induces apoptosis and suppresses tumor formation in a mouse AML xenotransplantation model (58). Stable SLC1A5 downregulation by a lentiviral approach inhibited human myeloma cell line growth *in vitro* and in a murine model (59). Activating mutations in NOTCH1 are common in T-ALL, and inhibition of NOTCH1 signaling in T-ALL drives a metabolic crisis, with prominent inhibition of glutaminolysis and promotes autophagy (60).

In addition, AML and ALL show dependence on arginine, and most AML and ALL cells lack arginine succinate synthase-1 (ASS1) and/or ornithine transcarbamylase (OTC), relying on extracellular arginine availability (61–63). AML constitutively expresses the cationic amino acid transporters CAT-1 and CAT-2B for arginine uptake, while ALL expresses CAT-1 in the absence of CAT-2A or CAT-2B (62, 63). BCT-100, a pegylated human recombinant arginase, leads to a rapid arginine depletion and could serve as a novel therapeutic agent for AML and ALL cells (62, 63). Arginine metabolism is significantly enriched in MM patients, promoting the urea cycle, and the elevated levels of urea, creatinine, and uric acid in plasma may be related to impaired renal function and damaged toxin excretion during the progression of MM (64, 65).

Indoleamine 2,3-dioxygenase (IDO) is an immunomodulatory enzyme that facilitates tryptophan catabolism into the immunoregulatory metabolite kynurenine (Kyn) (66). IDO and Kyn can manipulate the immunosuppressive tumor microenvironment by affecting T-cell maturation and proliferation and inducing differentiation into T regulatory cells (67). AML patients were shown to express IDO, and high IDO expression and elevated levels of Kyn were correlated with poor clinical outcomes in AML patients (68, 69). Inhibition of IDO expression can disrupt immune tolerance as an AML treatment option (68). Although individual CML patients differed in their rates of IDO production, the present data indicate that CML should be added to malignancies with higher IDO activity (70). CLL cells also express an active IDO enzyme and produce high levels of Kyn, which plays a role in the survival and drug resistance of leukemic cells (71). Another study demonstrates that the levels of serum Kyn and Trp are useful for predicting the prognosis of individual HL patients (72).

Asparagine is essential for DNA synthesis, RNA synthesis, protein metabolism, and survival of leukemic cells, however, ALL cells lack asparagine synthetase (ASNS). ALL cells are auxotrophic for asparagine and highly sensitive to asparaginase treatment (73, 74). Asparaginase depletes the source of asparagine for leukemic cells, leading to the death of leukemic cells, and the antileukemia effect has been shown in clinical treatment of ALL (75–77). Four drugs asparaginase *Erwinia chrysanthemi*, asparaginase *Escherichia coli*, calaspargase pegol, and pegaspargase, have been approved by the FDA to treat ALL, whereas pegaspargase was also feasible in higher-stage NHL (**Figure 2**).

Branched-chain amino acids (BCAAs) include leucine, isoleucine and valine, and branched-chain amino acids transaminases 1 (BCAT1) transfers α-amino groups from BCAAs to α-ketoglutarate (αKG) to produce glutamate and their respective branched chain ketoacids (BCKAs). BCAT1 is significantly overexpressed in AML leukemia stem cells (LSCs), resulting in enhanced α-KG amination and thus lowered intracellular levels of α-KG (78). BCAT1 is also aberrantly activated in CML, and blocking BCAT1 gene expression or enzymatic activity induces cellular differentiation and impairs the propagation of blast crisis CML (79).

### Lipid-Related Metabolism

Fatty acids (FAs) are key synthetic raw materials for cell membranes and important energy reserves. The oxidation and synthesis of FAs were shown to contribute to cancer growth. In CLL, STAT3 is constitutively activated, which also activates LPL transcription, resulting in elevated intracellular lipoprotein lipase levels (80). STAT3 also activates the fatty acid translocase CD36 and facilitates FAs uptake in CLL cells (81). LPL induces cellular uptake of lipoproteins, prompts the hydrolysis of triglycerides into free fatty acids (FFAs) and shifts CLL cell metabolism toward utilization of FFAs (82, 83). FFAs bind to proliferator-activated receptor (PPAR)-α as ligands, and the FFA-PPARα complex functions as a transcription factor to activate OXPHOS genes (84). The B-cell receptor (BCR) inhibitor ibrutinib could reduce LPL mRNA and protein levels and inhibit FFAs metabolism in CLL cells (85). Perhexiline inhibits carnitine palmitoyltransferases (CPT), thereby suppressing fatty acid transport into mitochondria and leading to massive CLL cell death (86).

Adipocytes could support cancer cells through the provision of FAs. ALL cells stimulate adipocytes lipolysis and take up FFAs released by adipocytes for OXPHOS (87). Adipocyte-derived FFAs can alleviate the dependence of ALL cells on *de novo* lipogenesis and reverse the cytotoxicity of pharmacological acetyl-CoA carboxylase (ACC) inhibition. In addition, the unsaturated fatty acid oleic acid protects ALL cells from modest concentrations of chemotherapy (87). Obesity was associated with worse outcomes and increased relapse rates in patients older than 10 years at ALL diagnosis (88). In addition to promoting fatty acid metabolism, MM cells induce lipolysis in bone marrow (BM) adipocytes and then take up the released FFAs through fatty acid transporter proteins (FATP), leading to growth (89). AML cells are also supplied free fatty acids from BM adipocytes, and utilize fatty acid oxidation (FAO) to generate energy (90).

Understanding the metabolic patterns of hematological malignancies will help us to better develop treatment plans for their metabolic changes. Metabolomics has rapidly begun to expand the research scope of genomics, transcriptomics, and proteomics. The comprehensive metabolic profiles offer a functional readout of cellular state, which sits closest in proximity to clinical phenotype. Interrogating metabolic rewiring of hematological malignancies at the single-celll resolution might help to elucidate the underlying causes of metabolic dysregulation in hematologic malignancy.

## Technical Advances in Single-Cell Metabolomics

Metabolomics has gradually exceeded the powers of genomics, transcriptomics, and proteomics to facilitate an understanding and assessment of the clinical phenotype. Numerous technologies and strategies are available for metabolism research. To determine what the individual cell is actually doing in nature, however, requires single-cell metabolomics. A broad array of new techniques allow researchers to catalog the chemical contents at single-cell resolution.

### Mass Spectrometry-Based Single-Cell Metabolomics Approaches

Mass spectrometry has emerged as the most widely used technique for single-cell metabolomics owing to its high sensitivity, broad molecular coverage, and wide dynamic ranges. MS is coupled with capillary electrophoresis (CE) and nano liquid chromatography (nanoLC), allowing efficient separation, sensitive detection, and identification of complex cellular contents (91–93). These hyphenated MS techniques enabled to identify hundreds to thousands of molecules with attomole to zeptomole sensitivity (94, 95). However, the application of these hyphenated MS techniques is limited by the relatively low throughput of cell analysis. Cell pretreatment inevitably causes strong cellular perturbation.

Mass spectrometry imaging (MSI) is an attractive approach to simultaneously image different compounds in a high-throughput manner, overcoming the limited number of molecules detected in traditional optical imaging (96, 97). Specially, MSI allows for visualization of the spatial distribution of biomolecules without extraction, purification, separation or labeling, which is in stark contrast to most label-based imaging methods.

The rapid development of single-cell metabolomics is attributed to single-cell separation and injection techniques, such as cell micro-array, single-cell droplet printing, and flow cytometry (93), that can then spawn new single-cell metabolomics technologies, such as high-density micro-arrays for mass spectrometry (MAMS), droplet-based electrospray ionization (ESI)-MS, and label-free mass cytometry (CyESI-MS). MSI has been coupled with many typical ionization techniques, such as matrix-assisted laser desorption ionization (MALDI), ion beam ionization, electrospray ionization, nanoelectrospray ionization (nESI), and matrix-free laser desorption ionization (LDI). MSI-based single-cell metabolomics technologies have evolved as the best suited platforms.

MALDI-MSI is one of the most popular techniques for single-cell metabolic analysis. With its minimal sample preparation and high throughput, MALDI–MS is well suited to analyzing large populations of cells, and has been used successfully to reveal cellular heterogeneity and to discover rare cell subtypes (98). High spatial resolution MALDI-MSI can achieve high-precision metabolite positioning at the cellular and subcellular levels *in situ*, which advances our understanding of complex biological processes by revealing unprecedented details of metabolic biology (99).

The direct injection of single cells separated by microfluidic devices or micropipettes into MS provides novel ways for highly sensitive metabolite analysis in single cells (100). Zhang et al. proposed a novel strategy integrating spiral inertial microfluidics and ion mobility mass spectrometry (IM-MS) for single-cell metabolite detection and identification, which offered a simple and efficient method for single-cell lipid profiling, with additional ion mobility separation of lipids significantly improving the confidence toward identification of metabolites (100).

### Single-Cell Metabolic Profiling by Flow Cytometry-Based Methods

With high sensitivity, broad molecular coverage, wide dynamic range, and structural identification capabilities, flow cytometry has become a widely used analytical tool for single-cell metabolomics. Met-Flow, a flow cytometry-based method, is capable of interrogating the network of metabolic pathways at the single-cell level within a heterogeneous population. Using Met-Flow, Patricia et al. captured the metabolic state of immune cells by targeting key proteins and rate-limiting enzymes across multiple pathways and discovered that glucose restriction and metabolic rewiring drive the expansion of an inflammatory central memory T cell subset (101).

Single-cell energetic metabolism by profiling translation inhibition (SCENITH) is a simple method for complex metabolic profiling samples *ex vivo*, that allows for the study of metabolic responses in multiple cell types in parallel by flow cytometry, particularly for rare cells. The ability of SCENITH to reveal global metabolic functions and determine complex and linked immune-phenotypes in rare cell subpopulations is helpful for evaluating therapeutic responses or patient stratification (102).

### Single-Cell Spatial Metabolomics

Dissection of spatiotemporal differences in metabolic activities of singular immune cells in the tumor microenvironment (TME) is the key to understanding their complex communication networks and the immune landscape that exists within compromised tissues. With its rapidly evolving methods, single-cell metabolomics technology is expanding to high spatio-temporal resolution, providing new platforms for spatial cell atlases and *in situ* visualization of metabolic processes.

High-spatial resolution MALDI-MSI has been applied to map and visualize the three-dimensional spatial distribution of phospholipid classes (103). Alexandrov et al. developed SpaceM, a method that integrates MALDI imaging with light microscopy and digital image processing to precisely match up the mass spectrometry data with the cells, which preserved the spatial relationships between the cells without requiring special preparation (104). The spatial single nuclear metabolomics (SEAM) method is a flexible platform combining high-spatial-resolution imaging mass spectrometry and a set of computational algorithms that can display multi-scale and multi-color tissue tomography together with the identification and clustering of single nuclei by their *in situ* metabolic fingerprints. SEAM is able to explore the spatial metabolic profile and tissue histology at the single-cell level, leading to a deeper understanding of tissue metabolic organization (105).

### Metabolic Modeling at the Single-Cell Level

Metabolic measurements at the single-cell level bring new insights into cellular function, which can often not be captured on other omics layers. However, single-cell metabolomics is limited by insufficient scalability and sensitivity, and is not yet widely available due to resource intensiveness. Metabolic modeling represents an interesting alternative strategy to infer latent cellular metabolism states from widely available information about reaction networks and other single-cell omics. Three main classes of modeling approaches used for prediction of metabolism on the single-cell level have been recently well reviewed, including pathway-level analysis, constraint-based modeling, and kinetic models (106). scMetNet constructs a metabolic network based on pathway repositories and then identifies metabolic rewiring across different cell populations (107). Single-cell flux estimation analysis (scFEA) infers the cell-wise fluxome from single-cell RNA-sequencing data by modeling the metabolic map with a graph neural network (108).

While an array of approaches for modeling cellular metabolic state have been proposed, a set of limitations restrict larger applicability. As experimental, technical, and biological reasons, intrinsic heterogeneity between different single-cell omics places a barrier to single-cell metabolic modeling from single-cell RNA-seq data. Single-cell proteomics measurements provide a promising alternative as they are more informative for prediction of enzyme activity. Modeling single-cell metabolism depends on well-established metabolic network models, which are basic to feasible metabolic conversions. Unpredictable metabolite exchanges between different cells and between individual cells and the environment pose additional challenges to modeling metabolism at the single-cell level. New metabolic modeling paradigms based on advanced computational approaches, such as deep learning, may enable efficient modeling at the single-cell and multi-omics levels.

### Single-Cell Multi-Omics

Single-cell multi-omics provides multiple biomolecular profilings of phenotypically heterogeneous cells on different biological layers. Multi-omics profiling of single-interacting cells in the native TME is essential for deeper understanding of the complex communication networks and the immune landscape that exist within compromised tissues. Tian et al. developed a new methodology called high-energy gas cluster ion beam-secondary ion mass spectrometry (GCIB–SIMS), which combined the chemical specificity of mass spectrometry with imaging resolution approaching 1 micron, small enough to image a single cell (109). GCIB–SIMS can comprehensively identify lipidomic and metabolomic profiling in different cell types, leading to new insights into the role of lipid reprogramming and metabolic response in normal regulation or pathogenic discoordination of cell-cell interactions in a variety of tissue microenvironments. Xiong et al. created a single-lysosome mass spectrometry (SLMS) platform that combined lysosomal patch-clamp recording with induced nanoelectrospray ionization mass spectrometry, which allowed the simultaneous detection of the electrophysiological properties and metabolome of the lysosome. These multimodal approaches open the door to much richer investigations into the interactions between cancer cells and immune cells, as well as cell–cell interactions in other systems (110).

## Application and Potential of Single-Cell Metabolomics in Hematopoiesis and Hematological Malignancies

### Tumor Biology

Metabolic reprogramming is one of the hallmarks of malignant tumors, which provides energy and material basis for tumor proliferation, invasion, metastasis as well as immune escape. Therefore, identifying the key metabolic factors that regulate cell cancerous changes and immune responses has become a major challenge. In recent years, single-cell metabolomics has emerged as a breakthrough technique that enable to directly measure metabolic states and defines unique cell types at unparalleled high resolution. Patricia et al. used Met-Flow to simultaneously measure divergent metabolic profiles and dynamic remodeling in human peripheral blood mononuclear cells and discovered that glucose restriction and metabolic remodeling drive the expansion of an inflammatory central memory T cell subset (101). Met-Flow is able to capture the complex metabolic state of individual hematopoietic cells, which will lead to a greater understanding of the role of metabolic reprogramming in hematopoiesis and hematological malignancies.

### Cellular Heterogeneity

Compositional heterogeneity is an inherent property of cell populations, presenting a major challenge in understanding the function of specific cellular subpopulations. Blocking the energy and material supply of tumor cells is one of the strategies for tumor treatment, however, metabolic heterogeneity of tumor cells hinders metabolic-based anti-tumor treatment. Metabolic differences can provide additional information to accurately identify cell state heterogeneity. To characterize cellular heterogeneity and discern specific subpopulations, molecular analysis at the single-cell level is necessary. Single-cell metabolomics analysis has revealed tremendous heterogeneity, conflicting with the classical view of hematopoiesis.

High-resolution mass spectrometry (HRMS) technology is an attractive approach to ultrasensitively detect proteins, peptides, and metabolites in limited amounts of samples, even single cells. Using single-cell capillary electrophoresis HRMS, Nemes et al. documented the differences in metabolite composition between left and right dorsal-animal blastomeres from the eight-cell frog embryo, indicating that metabolites trigger the differentiation of the stem cells into organ-specific lineages, and metabolite changes can alter cell fate (111). To extract trace-level signals from metabolic datasets with low abundances, the Trace framework was adopted, which incorporated machine learning (ML) to automate feature selection and optimization (112).

Precise discrimination of leukocyte subsets is very helpful for the clinical diagnosis of many diseases, especially for hematological malignancies. In order to rapidly discriminate various leukocyte subsets with specific functions, CyESI-MS was proposed to reveal leukocyte heterogeneity at the single-cell level. The single-cell metabolic fingerprints acquired by CyESI-MS as well as metabolite biomarkers can be used to distinguish different subtypes of leukemia cells from normal leukocytes, reflecting the application potential in clinical research (113).

### Immunometabolism

Metabolic reprogramming is vital for immune cell differentiation, function and fate (114, 115). The capacity of immune cells to respond to changing environments by metabolic reprogramming is crucial to their effector function. Single-cell metabolomics analysis offers robust solutions for profiling metabolites in a high-throughput manner and has substantially deepened our understanding of metabolic networks in immune cells. Cytometry by time of flight (CyTOF) platform uses metal-tagged antibodies to estimate the metabolic configurations within single cells and has largely expanded its capability in single-cell omics by combining additional markers, such as acetylation marks, metabolic signaling, and lineage markers. Levine et al. reported a CyTOF-based approach to define the metabolic features of CD8^+^ T cells in response to pathogen challenge at the single-cell level (116). This approach identified a transition state during an earlier stage of T-cell activation, characterized by high glycolytic and oxidative activity. Interestingly, analogous metabolic dynamics were observed in chimeric antigen receptor (CAR) T cells interrogated longitudinally in advanced lymphoma patients (116).

### Therapeutic Resistance

During tumor therapy, some patients develop therapeutic resistance, resulting in treatment failure and tumor recurrence. Metabolic rewiring is an effective way to evade immune cell antitumor activity, which is favored by cancer cells. Metabolic competition between tumor and immune cells limits nutrient availability and leads to microenvironmental acidosis in the tumor ecosystem, which hinders immune cell antitumor activity. By analyzing the genetic and metabolite information of individual cells, we can distinguish genes and regulatory pathways driving drug resistance development.

A wide multitude of research activities have been focused on immune evasion and drug resistance to overcome therapeutic resistance. Chen et al. utilized the single-probe mass spectrometry technique to analyze live irinotecan-resistant (IRI) cells under different treatment conditions, demonstrating a metformin-IRI synergistic effect overcoming drug resistance (117). Inhibition of fatty acid synthase (FASN) is a potential mechanism related to metformin treatment of drug-resistant cancer cells, which results in the downregulation of lipids and fatty acids. Liu et al. reported an analytical approach that combines single-cell mass spectrometry (SCMS)-based metabolomics with machine learning (ML) models to monitor the degree of drug resistance in early chemotherapeutic stage from single cells in their native microenvironment (118). This method can be potentially employed to evaluate chemotherapeutic efficacy in the clinic.

### Drug Development and Discovery

In recent decades, single-cell metabolomics has demonstrated enormous potential in many fields, including drug research and development. Current data suggest distinctive metabolic aberrations in hematological malignancies. Thus, molecular hallmarks of cancer cell metabolism provide opportunities for novel therapeutic interventions that will be complementary to existing diagnostic and treatment options. Preclinical or clinical trial studies using metabolic agents alone or in combination with other remedies have demonstrated promising outcomes. Numerous medications targeting cell metabolism are used in the care of patients with hematological malignancies (**Figure 2**), representing a promising endeavour in the search for effective treatment of hematological diseases.

Single-cell metabolomics promises to characterize metabolic reprogramming of cells in cancer, and shed light on metabolic effects of drugs (119). The application of single-cell metabolomics in drug discovery requires high throughput. Alexandrov et al. explored the potential of the recently developed method SpaceM for integration with high-content imaging and high-throughput applications in drug discovery, and successfully scaled up SpaceM to tens of samples (120).

Even though most of the findings on tumor metabolism derive from metabolomics analysis at the bulk level, single-cell metabolomics holds promise to further advance research on hematopoiesis and hematological malignancies. Given the ease of accessibility of liquid tumor biopsies in hematology, embedding single-cell techniques in routine laboratory diagnostics is feasible. Leveraging single-cell metabolomics to evaluate serial patient samples through diagnosis and the course of therapy provides a powerful means to stratify disease, evaluate tumor evolution, inform prognostication, and assist with treatment decisions. From this perspective, we foresee that single-cell metabolomics will boost the molecular diagnosis of hematological diseases, and open a new door to personalized medicine and the development of more effective therapies.

## Challenges and Perspectives of Single-Cell Metabolomics

Single-cell metabolomics is hitting its stride and is beginning to be widely employed to profile cellular metabolism. As we have shown in this review, single-cell metabolomics has many applications and enormous potentials, however, several limitations and challenges need to be further addressed. Firstly, sample preparation is a foundation for taking an accurate and complete snapshot of the metabolome. Due to rapid metabolic changes, sample preparation can alter the metabolome from its native state. Freeze-drying and chemical fixation are common methods for quenching metabolism, which allow some metabolites to diffuse away from the cell and eventually cause the loss of biological information. Different strategies, such as frozen hydration and capillary extraction, can be used to prepare samples for single-cell metabolomics analysis. Frozen hydration preserved the integrity and compartmentalization of the pristine molecular constituents of cells, reflecting a near-natural state of the metabolome (109). Capillary extraction efficiently separates the sample molecules with less damage to biological specimens than traditional dissection methods (111). Its resampling ability enables the combination of metabolomics with other omics techniques within the same cell, laying a foundation for obtaining single-cell multimodal profiles.

Secondly, the magnitudes of metabolite abundances vary wildly, emphasizing the need for single-cell metabolomics (SCM) techniques with ultrahigh sensitivity. Thus, heightening sensitivity is a common objective to maximize the chemical information obtained in single-cell metabolomics. For MS-based single-cell metabolomics, many efforts have been made to improve metabolite extraction, ionization techniques, and proprietary algorithms. A suitable extraction procedure is a prerequisite for detecting and identifying metabolites, especially for a tiny quantity of metabolites. Onjiko et al. employed capillary electrophoresis coupled with single-cell high-resolution mass spectrometry (CE-MS) to uncover small molecules (111). Then, Trace was used to extract trace-level signals to enhance sensitivity for metabolomics analysis (111). To achieve adequate sensitivity for metabolomics analysis, Takayuki et al. developed a “nanoCESI” emitter that allowed up to sub-nM detectability by establishing a reproducible fabrication process (95). Compared with a conventional sheathless emitter, the nanoCESI emitter improved the sensitivity by 3.5-fold, and by coupling with large-volume dual preconcentration by isotachophoresis and stacking (LDIS), further achieved up to 800-fold enhanced sensitivity. As new technologies and algorithms for single-cell metabolomics continue to develop and improve, researchers will be able to catalog the chemical contents of individual cells with high sensitivity.

The extreme complexity of the metabolome poses another challenge for single-cell metabolomics (121). The mass, charge, shape, and modification of metabolites must be considered in metabolite identification. Unpredictable new products generated by side reactions exacerbated this complexity. Another thorny issue is how to distinguish various isomers of large biomolecules. Definitively identifying and measuring the full and complete chemical contents from the cell is tricky. Spatial single-cell metabolomics allows hundreds of metabolites to be detected *in situ*. It is important to accurately match up metabolome data with the physical characteristics and neighborhood of the cell in the native context. The complexity of this analysis is sure to create new computational challenges. The integration of multidimensional data from single-cell omics is computationally challenging because of the intrinsic heterogeneity of these data (122). Fortunately, the recent explosion in molecular biology techniques and computational approaches makes it possible to overcome the above problems. Artificial intelligence (AI)-based methods have been successfully applied to several tasks of single-cell omics, representing powerful and promising tools for biological discovery (123). The power of single-cell metabolomics can be improved by combining artificial intelligence-based algorithms. New algorithms for metabolite identification and open-platforms will allow neophytes and seasoned investigators alike to make sense of the jumble peaks in the spectra, such as Trace (112) and METASPACE (124).

Finally, with respect to clinical applications, the clinical translation of single-cell metabolomics techniques is another key challenge in this field. Considering the complexity and sensitivity of SCM, the results derived from SCM must be interpreted with caution in clinical decisions. Additionally, the high cost of single-cell metabolomics creates a barrier to its widespread implementation in routine testing. With the advent of increasingly faster and cheaper high-throughput technologies, the integration of single-cell omics across modalities will expand our horizons and deepen our understanding of the interactions among the different biological layers. It is easy to imagine that single-cell methodologies will be applied routinely in clinical diagnostics, prognosis prediction as well as disease monitoring, which will revolutionize the diagnosis and therapy of patients.

## Author Contributions

FZ and XH contributed to investigation, data curation, illustration, writing, and editing. XH and JY contributed to writing-review and editing. All authors have read and agreed to the published version of the manuscript.

## Funding

This work was supported by (1) National Natural Science Foundation of China (No. 82172634 and 81902792); (2) Key Program of the Science and Technology Bureau of Sichuan (No. 2021YFSY0007); (3) 1.3.5 project for disciplines of excellence, West China Hospital, Sichuan University (No. ZYYC20013).

## Conflict of Interest

The authors declare that the research was conducted in the absence of any commercial or financial relationships that could be construed as a potential conflict of interest.

## Publisher’s Note

All claims expressed in this article are solely those of the authors and do not necessarily represent those of their affiliated organizations, or those of the publisher, the editors and the reviewers. Any product that may be evaluated in this article, or claim that may be made by its manufacturer, is not guaranteed or endorsed by the publisher.
